# Free-Living and Plankton-Associated Vibrios: Assessment in Ballast Water, Harbor Areas, and Coastal Ecosystems in Brazil

**DOI:** 10.3389/fmicb.2012.00443

**Published:** 2013-01-14

**Authors:** Irma N. G. Rivera, Keili M. C. Souza, Claudiana P. Souza, Rubens M. Lopes

**Affiliations:** ^1^Departamento de Microbiologia, Instituto de Ciências Biomédicas, Universidade de São PauloSão Paulo, Brazil; ^2^Departamento de Oceanografia Biológica, Instituto Oceanográfico, Universidade de São PauloSão Paulo, Brazil

**Keywords:** plankton-vibrio symbiosis, ballast water, port areas, bacterial hazard, *Vibrio cholerae*

## Abstract

Ballast water (BW) is a major transport vector of exotic aquatic species and pathogenic microorganisms. The wide-ranging spread of toxigenic *Vibrio cholerae* O1 from harbor areas has been frequently ascribed to discharge of contaminated BW into eutrophic coastal environments, such as during the onset of the seventh cholera pandemic in South America in the early 1990s. To determine the microbiological hazards of BWs transported to Brazilian ports, we evaluated water and plankton samples taken from (i) BW tanks of recently arrived ships, (ii) port areas along the Brazilian coastline from ∼1 to 32°S and (iii) three coastal areas in São Paulo State. *Vibrio* concentration and toxigenic *V. cholerae* O1 occurrence were analyzed. Plankton-associated vibrios were more abundant than free-living vibrios in all studied environments. *V. cholerae* was found in 9.5% of ballast tanks and 24.2% of port samples, both as free-living and attached forms and, apart from the Santos harbor, was absent off São Paulo State. Toxigenic *V. cholerae* O1 isolates (*ctx*A^+^, *tcp*A^+^), involved in cholera disease, were found in BW (2%) and harbor (2%) samples. These results confirm that BW is an important carrier of pathogenic organisms, and that monitoring of vibrios and other plankton-attached bacteria is of paramount importance in BW management programs.

## Introduction

Ballast water has been considered a principal transport vector of aquatic species of plants, animals, and microorganisms across biogeographic provinces, and to cause major changes in the composition and function of ecological communities in freshwater, estuarine, and marine ecosystems (Williams et al., 1988; Carlton and Geller, 1993; Ruiz et al., 2000). Ballast capacity varies according to vessel size and cargo type. A large commercial ship may carry an excess of 200,000 m^3^ of ballast water (BW), and discharge rates are as high as 20,000 m^3^ h^−1^ (NRC, 1996). Consequently, a large quantity and diversity of planktonic and benthic species occur in ballast tanks. Such species may succeed in transposing natural biogeographic barriers when viable individuals are released in the new environment through BW discharge, either in a single introduction event or as repeated inoculations (Williams et al., 1988; Carlton and Geller, 1993).

Bacteria belonging to the *Vibrionaceae* family, the so-called vibrios, are autochthonous from aquatic ecosystems worldwide and commonly found both as free-living cells and in association with plankton (Simidu et al., 1971; Kaneko and Colwell, 1975). Since the observation of *Vibrio cholerae* attached to copepod egg sacs and mouthparts (Huq et al., 1983), many studies have shown that vibrios are symbiotic to a wide range of zooplankton taxa (Louis et al., 2003; Rawlings et al., 2007; Lizarraga-Partida et al., 2009; Turner et al., 2009; Martinelli Filho et al., 2010).

Strong evidence exists that cargo ships are important transport vectors of cholera and other vibrio-related diseases (McCarthy and Khambaty, 1994; Ruiz et al., 2000; Drake et al., 2005, 2007; Mimura et al., 2005). Contaminated BW discharge into harbor and coastal waters could increase the likelihood of local horizontal gene transfer between toxigenic and non-toxigenic vibrio strains (Chiang and Mekalanos, 1999), thus setting the conditions for the spread of diarrheic outbreaks into a new location. This demands particular attention in coastal areas affected by impaired sanitary conditions, as in the case of many developing countries (Rivera et al., 2008). In addition, there is indication that anthropogenic climate change is driving the emergence of *Vibrio* disease in temperate regions (Baker-Austin et al., 2012). As a consequence, the spread of *Vibrio* species by maritime transport becomes a matter of public health concern even in countries where coastal pollution is a relatively minor environmental problem.

A significant percentage of cargo loads in Brazil is due to oil tankers and bulk carriers, which account for most of the BW transported globally. Loads moved by Brazilian ports and private terminals have more than doubled since the early 1990s, as a consequence of the country’s economical growth, and a corresponding increase in BW discharge by ocean-going ships is most likely underway (Oliveira, 2008). Our study covered the largest Brazilian harbors and some of the busiest coastal regions in terms of maritime transport.

We expand here the existing information on vibrios and *V. cholerae* prevalence in BW, harbor areas, and coastal regions, showing that toxigenic *V. cholerae* O1 occurs in ballast tanks, and that plankton-associated vibrios are two to four orders of magnitude more abundant than free-living vibrios, both in BW tanks and in the marine environment.

## Materials and Methods

### Ballast water and plankton samples from ships arriving to Brazilian ports

**Figure 1:**
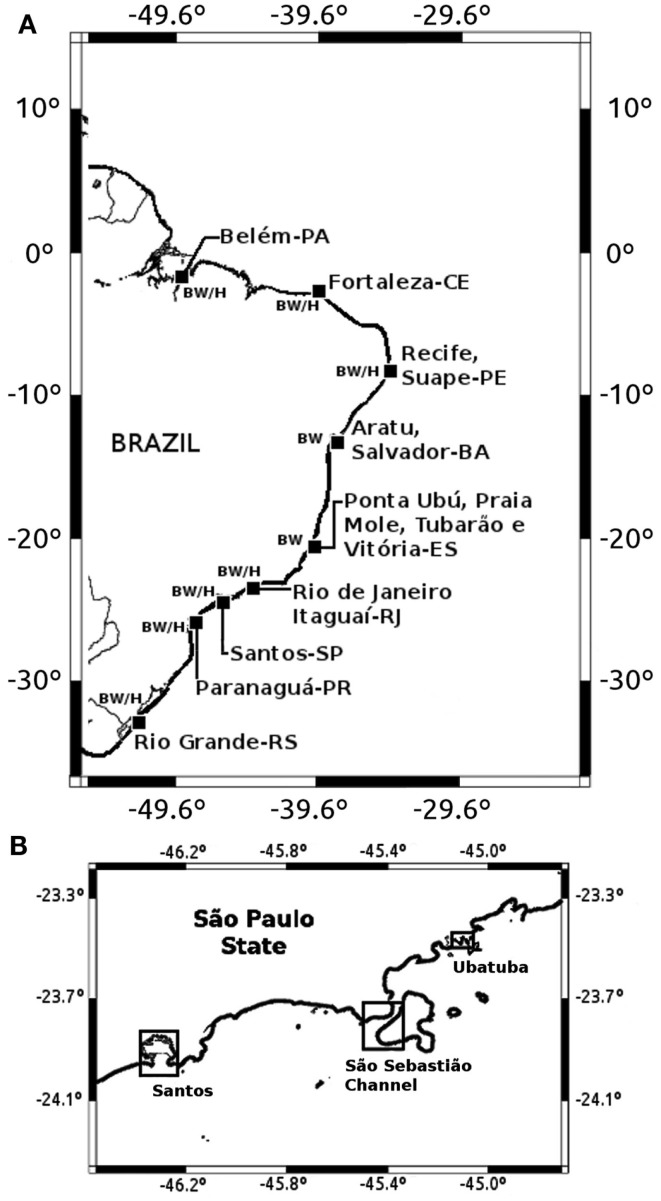
**Locations of ballast water (BW) and environmental (H) sampling sites in Brazilian ports (A) and in São Paulo State coastal areas (B)**.

Fifteen ports (Figure 1) were selected in nine Brazilian states based on their geographical representativeness along the extensive Brazilian coast, and according to operational characteristics such as prevailing navigation routes of arriving ships, ship types, and ship traffic volume. One hundred five commercial vessels were sampled covering both international and domestic routes (80 and 20% of samples, respectively). Using a suction pump, BW samples were collected from upper wing, fore peak, or double bottom tanks (one tank per vessel) accessed through sounding pipes, ellipses, scuttles, or vent pipes. One liter of BW was transferred to a sterile plastic bottle, after flushing at least 100 L of water from the pump hose. Pump pipes were abundantly rinsed and emptied after each use. Plankton samples were collected with the same pump by filtering 50–400 L of BW by wet-sieving through a 100-μm meshed-size conical net. Plankton samples were also transferred to 250 mL sterile plastic bottles. Sampling was done from October 2001 to 2002.

### Water and plankton samples from Brazilian harbor (H) and coastal areas

Seven Brazilian harbor areas were included in this study (Figure 1), each with six sampling stations along the harbor area and positioned from 40 m to 1 km off the main pier. Ninety water and 90 plankton samples were collected from October 2002 to April 2003 (Figure 1).

Three coastal regions in São Paulo state were additionally studied: Santos region (three stations), São Sebastião Channel (SSC; two stations), and Ubatuba (two stations; Figure 1). These stations/sites were selected according to their trophic status and level of anthropogenic influence. For instance, Santos is more urbanized and eutrophic than São Sebastião and Ubatuba (Burbano-Rosero et al., 2011).

Five liters of water were collected in a sterile plastic bottle during high tide for each station. Plankton samples were collected at the same locations by subsurface horizontal tows of a 64 μm mesh-sized net and transferred to a 250 mL sterile plastic container. In São Sebastião Channel, samples were collected monthly from August 2005 to March 2007 (20 months), while in Santos and Ubatuba sampling was performed during 2006 and 2007 summer seasons. A total of 32 seawater and 32 plankton samples were collected in São Sebastião; 15 seawater and 15 plankton samples in Santos; and 8 seawater and 8 plankton samples in Ubatuba (Figure 1).

### Environmental data

Salinity and temperature were measured in BW and environmental samples using a portable multi-probe (Hach Company). In order to summarize the salinity data, the water samples were classified in four categories: (1) oligohaline (less than 5 psu), poly/mesohaline (5–30 psu), euryhaline (30–35 psu), and oceanic (more than 35 psu).

We emphasized salinity as a main environmental variable in this study because ballast discharge regulations in Brazil are currently based on open-ocean exchange, which is validated by salinity inspection under the responsibility of port state control authorities.

Because sampling was performed in different periods of time between 2001 and 2007 for BW, harbor areas, and coastal regions of São Paulo state, correlations between vibrios and environmental data were performed separately according to the sample source. Zooplankton composition and biomass, as well as chlorophyll and other proxies for coastal eutrophication were not analyzed in this study.

### Sample transport to the laboratory

Immediately after sampling water and plankton samples were stored in an insulated container at 4°C and dispatched by courier or carried to the Environmental Microbiology Lab in São Paulo. Total time from sampling to analysis did not exceed 24 h.

### Marine vibrios counting

Concentration of viable vibrios was obtained with the plate count method associated with the Simidu and Tsukamoto (1980) medium. For plankton samples, 1 g (wet weight) was ground and three dilutions (1/10) were serially performed. The plates were incubated at 20°C for 72 h in anaerobic conditions (Anaerobac, Probac, SP, Brazil). *Vibrio* concentration was expressed as colony-forming units (CFU) mL^−1^ and CFU g^−1^ for water and plankton samples, respectively. Cultural determinations were based on duplicate plates at a given dilution, and results expressed as means. Considering that typical zooplankters such as calanoid copepods have a mean mass density only slightly higher than that of seawater (e.g., from 1.0274 to 1.0452 g cm^−3^ for *C. finmarchicus*; Knutsen et al., 2001), a direct comparison between free-living and attached bacterial concentrations in volumetric and mass terms is realistic for the purposes of this study.

### *Vibrio cholerae* detection and characterization

The enrichment method was used and water samples (100 mL) were filtered through 0.22 μm nitrocellulose membrane filters (Millipore), inoculated onto 25 mL of Alkaline Peptone Water (APW; 1% peptone; 1% NaCl; pH 8.4) and incubated at 30°C for 12–16 h. For plankton samples, 1 g (wet weight) was crushed, inoculated into 25 mL of APW and incubated under the same conditions. Two loops of the enrichment were streaked on thiosulfate-citrate-bile salts – TCBS Agar (Oxoid) and after incubation at 30°C for 18–24 h about five characteristic yellow colonies were transferred to Luria Agar (Difco). These colonies were screened for *V. cholerae* by the presumptive oxidase and string tests. The positive strains were preliminary screened by a short biochemical series (Choopun et al., 2002) and identified as *V. cholerae* by PCR (Chun et al., 1999). *V. cholerae* strains were serotyped by antiserum for O1 and O139 serogroups (Probac do Brasil). The serogroup was then confirmed by a multiplex PCR (Rivera et al., 2003). The detection of *ctx*A and *tcp*A genes, associated to virulence, was carried out by multiplex PCR using 94F and 614R primers for *ctx*A gene (Fields et al., 1992) and the 72F and 477R primers for *tcp*A gene (Rivera et al., 2001).

## Results

### Ballast water tanks

Free-living viable vibrios were more frequent (32%) than plankton-associated vibrios (18%) in BW samples but were not as abundant. Plankton-associated vibrios varied from <10 to 5,100 CFU g^−1^ while free-living vibrios varied from <1 to 430 CFU mL^−1^. Mean vibrio counts did not change significantly across the wide salinity gradient observed in BW tanks (Kruskal–Wallis test, *p* > 0.05). The temperature of BW samples varied from 19 to 35°C (mean 25.8°C) and the salinity varied widely, from 0.1 to 39.5 psu (mean 30.3).

*Vibrio cholerae* was detected in 13.3% of water and 5.7% of plankton samples from ballast tanks. *V. cholerae* O1 was isolated from eleven ballast tanks collected from ships arriving in Belém, Fortaleza, Recife, Santos, Itaguaí, Paranaguá, Ponta Ubú, and Rio Grande harbors (Table 1). Toxigenic *V. cholerae* O1, genotype *ctx*A^+^/*tcp*A^+^, was found in one water sample from a ship arriving in Belém and in one plankton sample from a ship arriving in Recife port. Two other BW samples contained *V. cholerae* O1, genotype *ctx*A^+^/*tcp*A^−^. Interestingly, *V. cholerae* non-O1 genotypes *ctx*A^+^/*tcp*A^+^ and *ctx*A^+^/*tcp*A^−^ were found in four other BW samples (Table 1).

**Table 1:** **Characteristics of *V. cholerae* strains isolated from ballast water tanks of ships arriving in Brazilian ports, with temperature and salinity records**.

Sample sequence and port identification	Ballast water		*Vibrio cholerae*		
	Temperature (°C)	Salinity (psu)	Free-living (F) or plankton-associated (A)	Serogroup	Genotype*
Belém	30	4.8	F	O1	*ctx*A^+^/*tcp*A^−^
Belém	32	29.8	F	O1	*ctx*A^+^/*tcp*A^+^
Belém	30	7.7	F	Non-O1	*ctx*A^+^/*tcp*A^+^
Fortaleza	28	9.7	F	O1	*ctx*A^−^/*tcp*A^−^
Recife	32	36.9	F	Non-O1	*ctx*A^+^/*tcp*A^−^
				O1	*ctx*A^+^/*tcp*A^−^
			A	O1	*ctx*A^+^/*tcp*A^+^
Recife	28	35.4	F	O1	*ctx*A^−^/*tcp*A^−^
			A	Non-O1	*ctx*A^−^/*tcp*A^−^
Ponta Ubu	20	34.4	F	O1	*ctx*A^+^/*tcp*A^−^
Ponta Ubu	22	34.6	F	Non-O1	*ctx*A^+^/*tcp*A^+^
Itaguaí	ND	34.3	F	Non-O1	*ctx*A^+^/*tcp*A^−^
Itaguaí	25	34.5	F	O1	*ctx*A^−^/*tcp*A^−^
Santos	29	35.3	F	O1	*ctx*A^−^/*tcp*A^−^
Santos	27	34.0	F	O1	*ctx*A^−^/*tcp*A^−^
			A	O1	*ctx*A^−^/*tcp*A^−^
Santos	27	34.5	F	O1	*ctx*A^−^/*tcp*A^−^
			A	O1	*ctx*A^−^/*tcp*A^−^
Santos	22	35.8	F	Non-O1	*ctx*A^+^/*tcp*A^+^
Paranaguá	26	34.9	A	O1	*ctx*A^−^/*tcp*A^−^
Rio Grande	19	33.4	A	Non-O1	*ctx*A^−^/*tcp*A^−^

*Genotypes *ctx*A and *tcp*A are genes codifying for cholera toxin and toxin co-regulated pilus, respectively. ND, not determined.

### Brazilian harbors areas (H)

**Figure 2:**
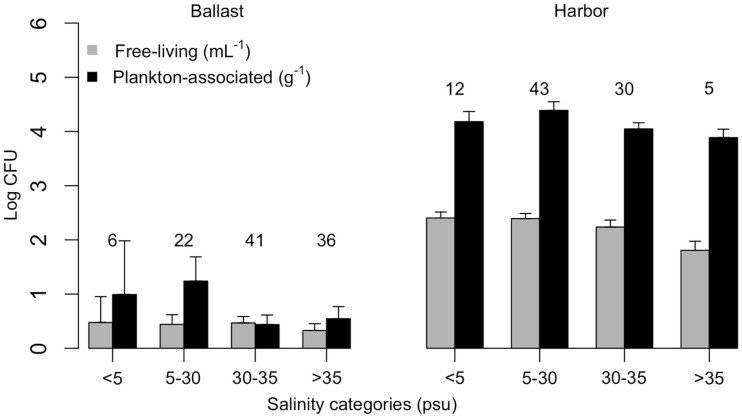
**Free-living (CFU mL^−1^) and plankton-associated (CFU g^−1^) vibrio concentration in ballast water (left panel) and harbor (right panel) samples, according to salinity categories**. Number of samples analyzed are indicated on top of standard error bars. CFU, colony-forming units.

Vibrios were prevalent in Brazilian harbor areas both as free-living (97%) and plankton-associated (96%) forms. Plankton-associated vibrios were more abundant (up to 2.4 × 10^6^ CFU g^−1^) than free-living (up to 4.4 × 10^3^ CFU mL^−1^; Table 2), and no relationship with salinity (Kruskal–Wallis test, *p* > 0.05) was observed in neither case (Figure 2). Temperature of harbor water samples varied from 16 to 35°C and salinity varied from 0.1 to 36.3 psu. The salinity values at the ports of Belém (PA) and Rio Grande (RS) were typical of freshwater and the remaining ports had estuarine characteristics.

**Table 2:** **Temperature, salinity, total coliforms, and vibrio frequency and concentration in environmental samples collected in Brazilian harbor areas**.

Ports	*n*	Temperature (°C)		Salinity		Total coliforms (CFU mL^−1^)		Free-living vibrios (CFU mL^−1^)			Plankton-associated vibrios (CFU g^−1^)		
		Min	Max	Min	Max	Min	Max	%	Min	Max	%	Min	Max
Belém	6	29	30	0	0.1	400	>2.0 × 10^4^	100	2.8 × 10^2^	1.6 × 10^3^	100	1.4 × 10^3^	8.6 × 10^4^
Fortaleza	6	27	27	27.3	35.9	<1	1.6 × 10^3^	100	2.4 × 10^1^	1.8 × 10^2^	100	1.7 × 10^3^	1.6 × 10^4^
Recife	24	23	29	26.2	34.9	<1	2.0 × 10^2^	100	4.2 × 10^1^	4.4 × 10^3^	91.7	<10	3.0 × 10^5^
Santos	24	21	27	10.8	32.5	<1	4.1 × 10^2^	96.7	<1	1.1 × 10^3^	100	2.0 × 10^2^	1.6 × 10^6^ψ
Paranaguá	18	16	35	16.8	27.4	16	9.2 × 10^2^	100	1.5 × 10^1^	3.3 × 10^3^	94.4	1.2 × 10^3^	2.4 × 10^6^ψ
Itaguaí	6	22	23	31.1	32.6	<1	<1	83.3	<1	1.7 × 10^2^	83.3	<10	7.7 × 10^3^
Rio Grande	6	20	21	0	0.1	<1	1.3 × 10^3^	100	5.8 × 10^1^	2.9 × 10^2^	100	3.2 × 10^3^	1.5 × 10^6^ψ

*The symbol ψ refers to estimated values*.

Both free-living and plankton-associated *V. cholerae* occurred in all harbor areas except Fortaleza and Rio Grande. Toxigenic *V. cholerae* O1 (*ctx*A^+^/*tcp*A^+^) isolates were found in water samples collected at Recife and Paranaguá, and in plankton samples collected at Santos harbor areas. Toxigenic *V. cholerae* non-O1 (*ctx*A^+^/*tcp*A^+^) was found as free-living forms in Belém, Recife, Santos, and Paranaguá harbors, and associated to plankton in the Recife and Santos harbor areas (Table 3).

**Table 3:** **Characterization of free-living and plankton-associated *Vibrio cholerae* strains isolated from environmental samples collected in Brazilian harbor areas**.

Harbor areas	*V. cholerae*		
	Free-living (F) or plankton-associated (A)	Serogroup	Genotype
Belém	F	O1	*ctx*A^−^/*tcp*A^−^
		Non-O1	*ctx*A^+^/*tcp*A^+^
Recife	F	O1	*ctx*A^+^/*tcp*A^+^, *ctx*A^+^/*tcp*A^−^
		Non-O1	*ctx*A^+^/*tcp*A^+^, *ctx*A^+^/*tcp*A^−^, *ctx*A^−^/*tcp*A^+^
	A	Non-O1	*ctx*A^+^/*tcp*A^+^, *ctx*A^+^/*tcp*A^−^, *ctx*A^−^/*tcp*A^+^
Itaguaí	F	Non-O1	*ctx*A^−^/*tcp*A^−^
Santos	F	O1	*ctx*A^+^/*tcp*A^−^
		Non-O1	*ctx*A^+^/*tcp*A^+^, *ctx*A^+^/*tcp*A^−^
	A	O1	*ctx*A^+^/*tcp*A^+^
		Non-O1	*ctx*A^+^/*tcp*A^+^, *ctx*A^+^/*tcp*A^−^
Paranaguá	F	O1	*ctx*A^+^/*tcp*A^+^
		Non-O1	*ctx*A^+^/*tcp*A^+^, *ctx*A^+^/*tcp*A^−^
	A	Non-O1	*ctx*A^−^/*tcp*A^−^

*Genotypes *ctx*A and *tcp*A are genes codifying for cholera toxin and toxin co-regulated pilus, respectively*.

### Coastal sites off São Paulo

**Figure 3:**
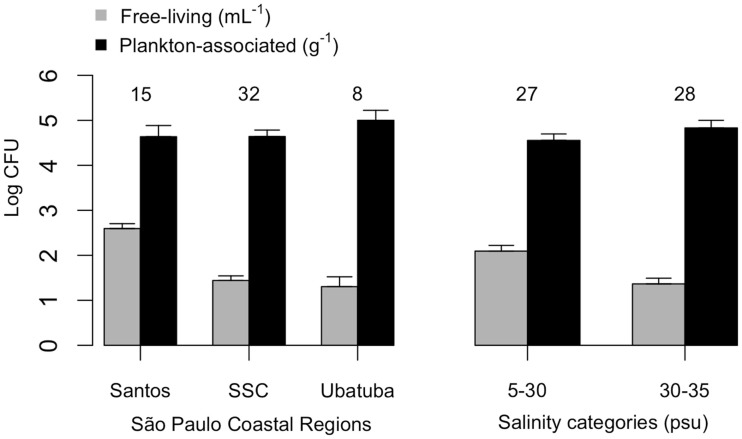
**Free-living (CFU mL^−1^) and plankton-associated (CFU g^−1^) vibrio concentration from São Paulo coastal areas**. Data shown by coastal site (left panel) and salinity categories (right panel). Number of samples analyzed are indicated on top of standard error bars. CFU, colony-forming units.

Mean plankton-associated viable vibrio abundance was again higher than free-living forms by two to four orders of magnitude (Table 4). Counts were higher for free-living vibrios in lower salinities (*p* < 0.001) but no difference occurred in the case of plankton-associated vibrios (*p* > 0.1). Mean free-living vibrio concentration was higher in Santos (*p* < 0.001) when compared with São Sebastião Channel and Ubatuba (Figure 3).

**Table 4:** ***Vibrio* frequency and concentration range on coastal areas of São Paulo State, Brazil**.

Coastal areas	Free-living vibrios (CFU mL^−1^)				Plankton-associated vibrios (CFU g^−1^)			
	*n*	%	Max	Min	*n*	%	Max	Min
Santos	15	100	1.6 × 10^3^	8.9 × 10^1^	15	100	1.8 × 10^6^	90
São Sebastião Channel	32	100	4.6 × 10^2^	2	32	100	1.4 × 10^6^	6.0 × 10^2^
Ubatuba	8	100	1.3 × 10^2^	2	8	100	8.0 × 10^5^	6.8 × 10^3^

*Vibrio cholerae* was not detected in coastal water or plankton samples with the traditional enrichment method applied here. From 110 collected samples, only 50 suspected colonies were obtained. However, when submitted to biochemical and molecular assays, those isolates were not confirmed as *V. cholerae*.

## Discussion

Cargo operations have a direct impact on BW management by commercial vessels, as BW is discharged to match a proportional amount of cargo being loaded. Using correlational methods based on such postulation, Medeiros (2004), Clarke et al. (2004), and Oliveira (2008) have shown that Brazilian harbors included in this study are recipients of BW imported from other biogeographic provinces.

Bacteria are known to attain high growth rates when associated to either live zooplankton or their carcasses, exuvia, and fecal pellets, as these substrates provide a much richer organic medium than the surrounding environment (Tang et al., 2010). Our results confirm such trend, as plankton-associated vibrios were two to four orders of magnitude more abundant than free-living bacteria in BW tanks, harbor areas, and coastal sites.

While associated vibrios were common in all aquatic ecosystems analyzed (>95% of samples), a much lower proportion of BW tanks contained either associated or free-living vibrios (18 and 32% of samples, respectively). Such low prevalence compared to the natural environment is probably related to increased zooplankton mortality in BW tanks (Gollasch et al., 2000) and the consequent loss of ambient chitin substrates for attachment, particularly in the case of chitinolitic bacteria such as vibrios. Dead zooplankton will eventually sink and accumulate in bottom sediments, and because zooplankton carcasses provide protection to bacteria from environmental stresses (Tang et al., 2010) it is likely that plankton-associated vibrios have been underestimated in BW tanks. A similar “protective refugia” may be found in biofilm matrices, which can sequester free-living bacteria during multiple fill and discharge cycles (Drake et al., 2005, 2007).

The same explanation may apply for the low detection rates observed for both associated and free-living *V. cholerae* O1 and non-O1 in BW samples. *V. cholerae* O1 often appears in the so-called viable but non-culturable (VBNC) form, requiring immunological and molecular tests for detection (Colwell and Huq, 1994; Huq et al., 2000). For instance, the use of direct immunofluorescence microscopy has yielded high detection rates for *V. cholerae* O1 in estuarine and coastal sites in Brazil (67–90%, Martins et al., 1993; Martinelli Filho et al., 2010) and in ship’s BW in the United States (Ruiz et al., 2000).

*Vibrio* assessment in harbor and coastal sites is critical to evaluate the microbiological risk of BW discharges and to provide a baseline for BW management and surveillance programs. Cholera and toxigenic *V. cholerae* O1 offer a useful model to examine the transportation of pathogenic microorganisms by BW (Ruiz et al., 2000). This association has essentially emerged from the recognition that BW played a role in the spread of the seventh cholera pandemic in South America in the early 1990s (Colwell, 1996). The environmental constraints in cholera epidemics have also been emphasized by Colwell (1996) who suggested that peaks in vibrio abundance were linked to the increase in copepod production during El Nino event. Additionally, Baker-Austin et al. (2012) reported the association of warming patterns to emergence of *Vibrio* infections in the Baltic area.

Contrasts in the aquatic ecosystem between donor and receiving regions are important in defining the likelihood of a successful invasion, including that of an emerging disease. Our finding of a toxigenic *V. cholerae* O1 strain (*ctx*A^+^, *tcp*A^+^) in BW from a ship that had just arrived in the Belém harbor yields a good example: the toxigenic bacteria thrived in a much more saline environment within the tank (salinity of 29.8 psu) compared to the surrounding environment, as Belém is a freshwater harbor. This most likely helped to prevent the arriving toxigenic strain from establishing itself in that location.

Nevertheless, the presence of toxigenic *V. cholerae* O1 in BW tanks from ships arriving in Brazilian ports, as well as in local harbor areas, is a matter of concern. We detected toxigenic (*ctx*A^+^, *tcp*A^+^) *V. cholerae* non-O1 strains, which potentially interact with naturally occurring non-toxigenic serogroup O1 or may convert itself to *V. cholerae* O1 by conjugation, or seroconversion (Chiang and Mekalanos, 1999). The frequency of environmental strains of non-O1 *V. cholerae* containing virulence-associated factors is low (Rivera et al., 2001; Vital Brazil et al., 2002), however the emergence of *V. cholerae* O1 toxigenic strains (*ctx*A^+^) resulting from lysogenic infection and conversion by the filamentous phage CTXΦ (Waldor and Mekalanos, 1996) and other genetic virulence elements has been described (Karaolis and Kaper, 1999). Once introduced in the environment by contaminated BW discharge, a toxigenic population of *V. cholerae* O1 may genetically interact with a non-toxigenic native population setting conditions for a cholera outbreak, especially where sanitary conditions are poor. An invasive *V. cholerae* strain could then be further transported by ships to other regions or dispersed naturally by aquatic currents, thereby affecting a large geographic region. The role of coastal eutrophication on vibrio distribution was depicted in our study by the high vibrio abundances found in the Santos area, where poor water management practices prevail.

The use of *V. cholerae* as a model pathogenic bacterium in BW analysis is a crucial approach to ensure the sanitary quality of coastal waters and to prevent the spread of cholera epidemics by maritime transport. Long-term BW surveillance programs will certainly help to minimize the introduction of toxigenic *V. cholerae* into new areas. For that purpose, methods to differentiate pathogenic from non-pathogenic *V. cholerae* populations in aquatic ecosystems have been proposed and successfully implemented (Rivera et al., 1995, 2003), and recent achievements in microbial detection using lab-on-a-chip approaches (e.g., Jung et al., 2011) may prove extremely useful for BW monitoring applications.

## Conclusion

Despite research and management initiatives carried out by a number of organizations worldwide, shipping continues to represent a threat as a major vector for the transfer of invasive aquatic species. A considerable effort has been given to the study and control of non-indigenous plants, algae, and invertebrates transported by cargo ships, but fewer investigations exist on the role of BW discharge in the spread of bacteria across biogeographical provinces and in the dissemination of emerging aquatic diseases. We believe these studies are essential, and strongly encourage the engagement of microbiologists, plankton ecologists, and engineers in the search for novel solutions for BW monitoring systems. Future developments in fast and reliable detection techniques are essential to implement cost-effective and environmentally sound BW management programs.

## Conflict of Interest Statement

The authors declare that the research was conducted in the absence of any commercial or financial relationships that could be construed as a potential conflict of interest.
